# Therapeutic and Mechanistic Perspectives of Protein Complexes in Breast Cancer

**DOI:** 10.3389/fcell.2019.00335

**Published:** 2019-12-20

**Authors:** Mark P. Waterhouse, Rosie Ugur, Walid T. Khaled

**Affiliations:** Department of Pharmacology, University of Cambridge, Cambridge, United Kingdom

**Keywords:** transcription factor, breast cancer, TNBC, protein-protein interaction, protein complexes, cancer therapy, PROTAC, post-translational modification

## Abstract

Breast cancer affects one in eight women making it the most common cancer in the United Kingdom, accounting for 15% of all new cancer cases. One of the main challenges in treating breast cancer is the heterogeneous nature of the disease. At present, targeted therapies are available for hormone receptor- and HER2-positive tumors. However, no targeted therapies are currently available for patients with triple negative breast cancer (TNBC). This likely contributes to the poor prognostic outcome for TNBC patients. Consequently, there is a clear clinical need for the development of novel drugs that efficiently target TNBC. Extensive genomic and transcriptomic characterization of TNBC has in recent years identified a plethora of putative oncogenes. However, these driver oncogenes are often critical in other cell types and/or transcription factors making them very difficult to target directly. Therefore, other approaches may be required for developing novel therapeutics that fully exploit the specific functions of TNBC oncogenes in tumor cells. Here, we will argue that more research is needed to identify the protein-protein interactions of TNBC oncogenes as a means for (a) mechanistically understanding the biological function of these oncogenes in TNBC and (b) providing novel therapeutic targets that can be exploited for selectively inhibiting the oncogenic roles of TNBC oncogenes in cancer cells, whilst sparing normal healthy cells.

## Breast Cancer Subtypes and Associated Therapies

Breast cancer is the most common cancer in the United Kingdom, accounting for 15% of all new cancer cases, and is the leading cause of cancer death in women worldwide (Bray et al., 2018). Historically, breast cancers have been classified based on the expression of several cell-surface receptors, namely the estrogen receptor (ER), progesterone receptor (PR), and human epidermal growth factor receptor 2 (HER2) (Onitilo et al., 2009). Based on the presence or absence of these markers, breast cancers can be broadly stratified into luminal A/B, HER2+, or basal-like (triple-negative) subtypes. Specifically, luminal A/B breast cancers are characterized as hormone receptor positive (high expression of ER and/or PR); HER2+ breast cancers are characterized by amplification of HER2 (and can be ER+ and/or PR+); and triple negative breast cancers (TNBCs) are characterized as hormone receptor negative and HER2 negative (ER−/PR−/HER2−) (Onitilo et al., 2009). Receptor status continues to act as a critical assessment for all breast cancers, likely due to the quick, easy and cost-effective stratification of patients to determine suitability for targeted treatments. These include tamoxifen, an ER modulator, and trastuzumab (Herceptin), a monoclonal antibody targeting the HER2 receptor, which are first-line therapies for ER+ tumors in pre-menopausal women and HER2+ tumors, respectively. Due to the availability of effective treatment options, hormone receptor positive breast cancers generally have a better prognosis (Fallahpour et al., 2017). Prior to the advent of modern therapies, HER2+ patients had a worse prognosis. However, since the introduction of HER2-targeted therapies, such as trastuzumab, there has been a significant improvement in prognosis (Slamon et al., 2001). Conversely, TNBCs (i.e., negative for all three hormone receptors) still lack targeted treatments and continue to have a comparatively poor prognosis (Dent et al., 2007; Onitilo et al., 2009).

**FIGURE 1:**
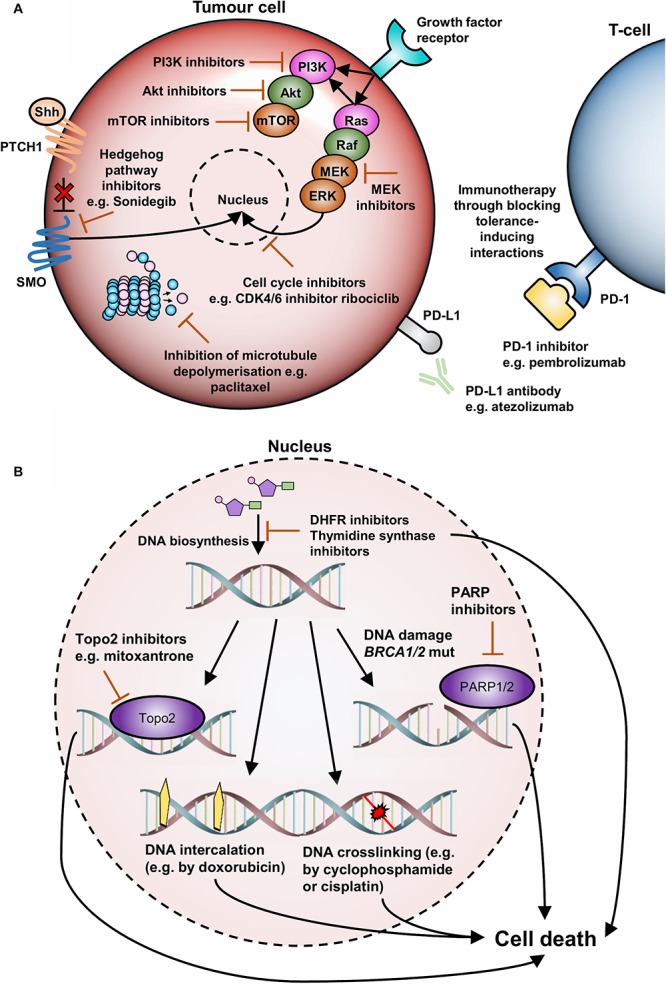
An overview of current and emerging agents for TNBC therapy. **(A)** TNBC therapies targeting the cell surface and cytoplasm. Cell surface therapies include inhibitors of immune tolerance inducing proteins such as PD1 and PD-L1. Cytoplasmic therapies include inhibitors of the Ras/MAPK pathway, especially MEK inhibitors, inhibitors of the PI3K/AKT/mTOR pathway, inhibitors of the Hedgehog signaling pathway, and cell cycle inhibitors such as paclitaxel and CDK inhibitors. **(B)** TNBC therapies targeting the nucleus. These therapies tend to target DNA synthesis and repair pathways or affect DNA viability to induce cell-cycle arrest and cell death.

For tumors that are susceptible to targeted therapies various therapies are available (Lumachi et al., 2013; Fallahpour et al., 2017). For hormone receptor-positive cancers, these include selective ER modulators, inhibitors of the aromatase enzyme and antigonadotropic therapies. Patients with HER2+ tumors also benefit from the availability of the monoclonal antibodies trastuzumab and pertuzumab, which function by preventing HER2 from functioning by inhibiting HER2-associated signaling (Molina et al., 2001; Agus et al., 2002; Franklin et al., 2004; Junttila et al., 2009). For tumors that are unsuitable for targeted therapies (i.e., TNBC), treatment involves chemotherapy in combination with radiotherapy and/or surgery (Foulkes et al., 2010; Wahba and El-Hadaad, 2015). Chemotherapeutic agents that are currently approved for use in breast cancer therapy typically target DNA synthesis and repair pathways and tend to have more serious side effects. Mechanistically, these therapies comprise alkylating agents that irreversibly crosslink DNA and lead to apoptosis (Hall and Tilby, 1992) [cyclophosphamide, mitomycin C (Tomasz, 1995)]; inhibitors of DNA biosynthesis enzymes such as dihydrofolate reductase [methotrexate (Goodsell, 1999)], thymidine synthase [fluorouracil (Longley et al., 2003)] and type II topoisomerase [mitoxantrone (Fox, 2004)]; or DNA intercalators that inhibit DNA and RNA synthesis [epirubicin, doxorubicin (Gewirtz, 1999)] (Figure 1A). Cytoskeletal drugs are also approved for use in breast cancer therapy (e.g., paclitaxel) and block cell cycle progression by stabilizing microtubule polymers (Horwitz, 1994; Figure 1B).

In hormone receptor and/or HER2+ positive tumors, it appears that much efficacy is derived from the availability of therapies targeting protein-protein interactions (PPIs) that drive disease. These therapies, which include tamoxifen, anastrozole and trastuzumab, either directly or indirectly block interactions between growth factors and their receptors. However, due to the lack of actionable receptors in TNBC, chemotherapy remains the first-line standard of care in combination with radiotherapy and/or surgery. It is apparent that these non-targeted chemotherapeutic agents represent generic therapeutic strategies that broadly target cancerous tissues, as they preferentially target rapidly dividing cells such as those found in tumors. However, normal cells that divide rapidly such as those in the digestive tract, hair follicles, and bone marrow are also highly susceptible to cytotoxicity, which leads to common chemotherapeutic side effects such as mucositis, alopecia, and myelosuppression with subsequent immunosuppression (Partridge et al., 2001). As a result, there is a clear clinical need for the identification of actionable targets in TNBC that can be used as the basis for the production of new and more targeted therapies. It is likely that, to ensure specificity in targeting, the identification of PPIs or protein networks that drive disease will be necessary for this purpose.

A promising example of this concept is the inhibition of interactions that induce immune tolerance such as the PD-1/PD-L1 axis (Gatalica et al., 2014), inhibition of which promotes T-cell proliferation, survival and cytotoxicity (Figure 1A). Most recently this has included the approval of atezolizumab, an anti-PD-L1 antibody, in combination with chemotherapy for the treatment of PD-L1+ metastatic TNBC by the US FDA in March 2019 (Schmid et al., 2018; Dolgin, 2019). Another example is the recent use of poly ADP ribose polymerase (PARP) inhibitors for the treatment of *BRCA1*-mutated tumors. Although there continues to be a lack of targeted treatment options for TNBC patients, ∼15% of TNBC tumors are driven by germline mutations within the *BRCA1* and *BRCA2* genes (Engel et al., 2018). These mutations result in defective double-strand DNA repair machinery and lead to the accumulation of DNA damage. PARP is another DNA repair protein that is crucial for the repair of single-strand DNA breaks (Audebert et al., 2004; Heale et al., 2006), which can develop into double-strand breaks (DSBs) if not repaired before the initiation of DNA replication (Farmer et al., 2005). In this context, *BRCA1/BRCA2* mutated tumors cannot repair these DSBs, ultimately resulting in cell death, whereas normal cells can compensate for the loss of PARP function (Farmer et al., 2005). As a result, patients with mutated *BRCA1/BRCA2* are suitable candidates for additional treatment with PARP inhibitors, such as the recently approved drug olaparib which was approved in 2019 in Europe for germline *BRCA1/2*-mutated HER2− breast cancer (Griguolo et al., 2018; Le and Gelmon, 2018). However, this therapy class is only suitable for patients with *BRCA*-mutated tumors and there is still intense interest in the identification of the molecular drivers of TNBC.

## Molecular Profiling of Breast Cancers for Target Identification

Much effort has been invested into the molecular profiling of breast cancers for the identification of novel drivers in TNBC pathogenesis and to better define breast cancer subtypes. The first of these classification models, proposed by Sørlie et al. (2003), was based on the transcriptomic profiling of 115 malignant breast tumors and identified five intrinsic subtypes of breast cancers (Sørlie et al., 2001, 2003). Although the identification of these intrinsic subtypes has provided much insight into breast cancer biology, attempts to define possible somatic drivers of breast cancer subtypes has remained difficult due to the heterogeneity of the disease as well as a lack of clear driver mutations. More recent work has aimed to tackle this issue by integrating genomic and transcriptomic breast cancer data in much larger patient sizes, a prime example of which is the recent METABRIC dataset. This work characterized the genomic and transcriptomic architecture of 2000 breast tumors (Curtis et al., 2012) and resulted in the identification of 10 novel molecular subgroups, known as integrative clusters, which are clustered according to copy number alterations and gene expression data (Dawson et al., 2013). Crucially, each integrative cluster is associated with distinct clinical features and outcomes (Dawson et al., 2013). In addition, the clusters have identified heterogeneity within tumors classified according to receptor status and divided all previously identified intrinsic subtypes into separate groups. Additional transcriptomic studies have further highlighted the heterogeneity of TNBC, which include studies by Lehmann et al. (2011, 2016) and Burstein et al. (2015), both of which identified four molecular subtypes of TNBC. As a result, breast cancer classification is now evolving to describe a number of distinct molecular subgroups based on multiple genomic factors, which has produced more robust patient classifiers and is leading to a new stratification and treatment paradigm for breast cancer patients. However, despite this progress in the molecular characterization of TNBC, these tumors remain to be mostly characterized by *TP53* alterations and copy number alterations involving 5q loss and gains at 8q, 10p and 12p (Dawson et al., 2013).

A limited number of studies have therefore attempted to investigate the mutational landscape in TNBC, which has mostly identified that TNBC is characterized by a low rate of activating point mutations in common oncogenes, as well as extensive individually rare mutations in other genes (Shi et al., 2018). However, TNBCs appear to be particularly enriched for alterations in tumor suppressor proteins, such as *TP53*, *RB1*, and *PTEN*, as well as oncogenic alterations in the PI3K/AKT pathway (Curtis et al., 2012; Koboldt et al., 2012). Regardless, common TNBC “oncogenes” such as *PIK3CA* and other actionable targets, such as the Ras/MAPK (Balko et al., 2013), JAK/STAT (Marotta et al., 2011), Wnt (DiMeo et al., 2009), TGF-β (Bhola et al., 2013), Hedgehog (Liu et al., 2006), and Notch (Harrison et al., 2010) pathways, are all critical genes/signaling pathways in a wide range cell types and contexts. As a result, any therapies designed against these pathways are highly likely to result in off-target cytotoxicity. Overall therefore, genome-wide studies have failed to identify driving mutations distinct from those affecting TP53, PIK3CA, and PTEN (Peluffo et al., 2019), and new therapeutic angles are required to define better and more specific targets for the production of TNBC therapies. One such angle to consider is that alterations in epigenetics and transcriptional machinery may be largely contributing to the transcriptional dysregulation seen in TNBC malignancies.

## Transcription Factor Targeting for Potential Enhanced Therapeutic Specificity

Downstream effectors of traditionally targeted pathways, namely transcription factors (TFs) involved in normal cellular function, are often those subjected to dysregulation resulting in cancer (Bass et al., 2009). Indeed, many cancer-related events either directly involve TFs or indirectly modulate TF activity. This highlights targeting TFs as a promising anticancer strategy and as potentially superior therapeutic targets compared to upstream signaling proteins and kinases (Konstantinopoulos and Papavassiliou, 2011). Our progression in understanding of the mechanistic properties of TFs and their associated networks, in both diseased and normal cells, has created huge potential for precision medicine in cancer. For example, targeting oncogenic TFs may lead to preferential cancer cell death in tumors that display TF dependency, whereas normal cells may be more likely to tolerate a loss of TF function due to redundancies in normal signaling pathways. One such case is the *TRPS1* TF, which demonstrates breast lineage-specific transcriptional dependency, likely due to lineage-restricted expression (Witwicki et al., 2018). As a result, breast cancer cells lines display sensitivity to *TRPS1* shRNA targeting compared to cell lines derived from colon, neuroblastoma, leukemia, prostate, and rhabdoid tumors (Witwicki et al., 2018). TFs in this context are therefore likely to have a high therapeutic potential, owing to their critical role in tumor pathogenesis along with their dispensability for physiologic cell function. Accordingly, many studies have tried to capture the transcriptional landscape of TNBC, thus identifying highly expressed genes and TFs that may be liable to therapeutic targeting. However, TFs have long been considered “undruggable” targets, which may result from the large interaction surface areas used by TFs for protein-DNA and PPIs as well as their predominant nuclear localization, which makes them less accessible to therapeutic agents (Yan and Higgins, 2013).

**FIGURE 2:**
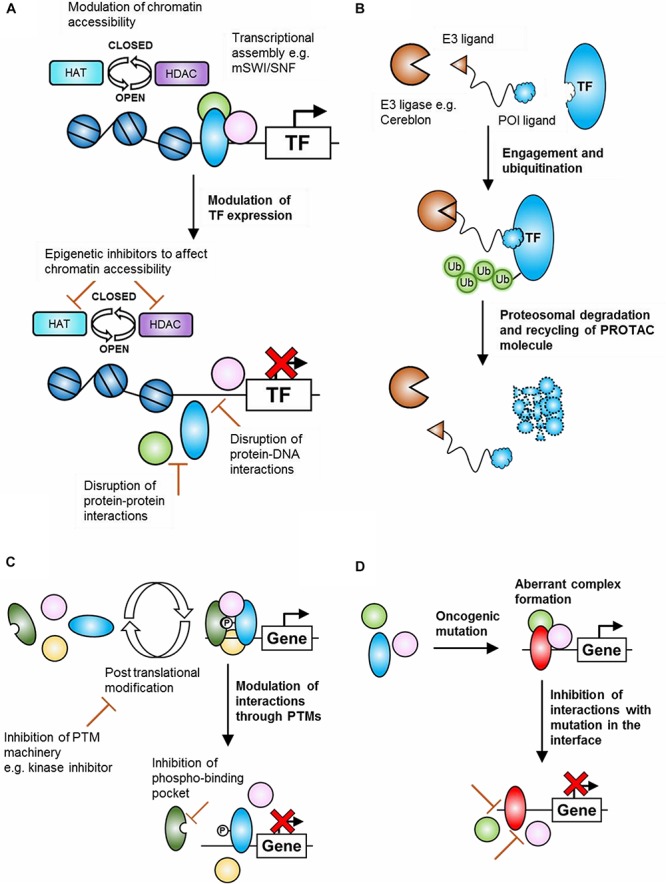
Potential mechanisms of transcription factor (TF) targeting for cancer therapy. **(A)** Inhibition of oncogenic TF expression. This may take the form of altering chromatin accessibility, through inhibitors of epigenetic machinery, or by disrupting the assembly of transcriptional machinery at the protein-protein or protein-DNA binding level. **(B)** Depletion of oncogenic proteins by PROTAC-mediated proteasomal degradation. A bi-functional molecule containing a protein of interest-binding region and an E3-ligase binding region links the protein of interest to an E3-ligase, leading to ubiquitination and proteasomal degradation. **(C)** Inhibition of TF function through modulation of post-translational modifications. Assembly of oncogenic transcriptional (or epigenetic) assemblies may rely on post-translational modifications. Inhibiting the enzymes responsible for these modifications or inhibiting the binding pocket of the specific modification may represent feasible options for preventing the assembly of oncogenic transcriptional assemblies. **(D)** Inhibition of mutation-dependent transcriptional assemblies. Structural information regarding the binding interfaces of mutated transcriptional or epigenetic proteins may allow for the design of therapies that inhibit mutation-dependent interactions and prevent the assembly of mutation-dependent transcriptional machinery.

Despite these challenges, there are various opportunities available for targeting TFs at different functional levels. For example, TFs may be directly or indirectly targeted through inhibition (or activation) at the expression level, at the PPI level, at the post-translational modification level, at the protein/DNA binding level, through the binding of a small molecule in an inhibition/activation pocket or through physical degradation (Figure 2). In addition, post-translational modifications, which may result in context-specific PPIs and/or differential assembly of epigenetic remodeling complexes, must also be considered. To date, over 450 unique protein modifications have been described, including phosphorylation, acetylation, ubiquitination, methylation, and SUMOylation, which can alter target protein activity, intracellular distribution, protein interactions and protein longevity (Venne et al., 2014). For phosphorylation alone, there are over 500 different kinases in mammals (Woolfrey and Dell’Acqua, 2015), some of which could conceivably be expressed in a tissue-specific manner and may therefore give rise to differing versions of the same proteins in various tissues.

Like all other cellular proteins, TF expression is controlled by transcriptional activators and repressors (such as other TFs or itself in a feedback loop) as well as by epigenetic machinery. Aberrant activity of these processes may therefore result in oncogenic transcriptional programs. For example, oncogenic gene translocation and consequent juxtaposition of the c-MYC gene with enhancer elements has been reported in multiple myeloma, which may enhance c-MYC expression (Shou et al., 2000), and aberrant expression of the HOXA cluster of TFs has been reported in several aggressive acute leukemias as a result of oncogenic rearrangements of the *MLL1* gene, a histone methyltransferase (Kawagoe et al., 1999; Guenther et al., 2005). More specifically, rearrangements of the *MLL1* gene can lead to the production of over 70 in-frame oncogenic fusion proteins, which can add functionality by enabling interactions with histone methyltrasferases such as DOT1L (Krivtsov et al., 2007) or may direct the MLL complex to unintended genomic areas, resulting in an aberrant transcriptional program. Therapies have been designed for both of these contexts, typically through regulation at the epigenetic level. For example, HDAC and histone methyltransferase inhibitors (e.g., against DOT1L), both of which associate with the MLL complex, have entered clinical trials for acute myeloid leukemia (Daigle et al., 2011; Chen et al., 2013; Fredly et al., 2013; Morabito et al., 2016), whereas negative regulation of oncogenic c-MYC has been achieved, for example, by inhibitors of BRD4 (such as JQ1) which displace BRD4 from the c-MYC promoter (Fowler et al., 2014). Control of HOXA expression has also been attempted through disruption of the MLL complex, for example by inhibiting the incorporation of WDR5 into the MLL complex which is required for the enzymatic activity of MLL1 (Li et al., 2016; Karatas et al., 2017). These examples represent indirect TF targeting at the epigenetic and PPI levels.

As well as indirect inhibition of TF function, direct inhibition of TF interactions may be an attractive therapeutic approach. Targets in this case may include single TF homodimers, a specific heterodimeric TF pair or a multimeric transcriptional complex. Indeed, TFs have the potential to form a large number of dimeric structures with distinct biological properties (over 500 dimers in human and up to 2500 dimers when considering alternate splicing) that can allow for elaborate fine-tuning of responses (Amoutzias et al., 2008). The concentration of each monomer in the cell, its post-translational modifications and its binding affinity for other monomers all play a role in determining dimer formation and, consequently, will determine which signaling process will dominate. These regulatory mechanisms therefore offer multiple levels of complexity and likely represent an underexploited therapeutic opportunity, as targeting specific TF dimers or specifically modified TFs (e.g., phosphorylated at a specific position) may offer exquisite therapeutic specificity. This may become a viable approach through the identification of TF states that contribute toward disease pathogenesis, especially as TFs have traditionally been considered undruggable.

A good example of this concept is the Myc-Max and Mad-Max heterodimerization system, whereby Max is a ubiquitous protein that can heterodimerize with either Myc or Mad (Grandori et al., 2000; Lüscher, 2001). Similarly, Myc and Mad can only heterodimerize with Max, but not each other. Upon formation of the Myc-Max heterodimer, recruitment of the mSWI/SNF nucleosome remodeling complex or histone acetyltransferases (HATs) occurs at the promoters of target genes, resulting in transcriptional activation. Conversely, formation of the Max-Mad heterodimer leads to recruitment of HDACs, which results in silencing of target genes and is antagonistic to the Myc-Max heterodimer. As a result, variations in the concentrations or affinity of these complexes can lead to a transcriptional bias and potentially alter the oncogenic capacity of the cell. As previously discussed, post-translational modifications may offer an additional level of complexity and can determine the transition to a functional dimeric TF pair. This has been observed through the phosphorylation of STAT proteins (Levy and Darnell, 2002) as well as ReIB, which leads to the formation of p100-ReIB dimers (Maier et al., 2003). Another example is the phosphorylation of the bHLH protein E47, which blocks formation of the homodimer and favors formation of a heterodimer with MyoD, leading to the activation of muscle-specific transcriptional activity (Lluís et al., 2005). It is therefore feasible to suggest that phosphorylated versions of TFs that participate in oncogenic interactions or transcriptional programs may represent attractive therapeutic targets in the future, especially if the phosphorylated protein does not exist or is very rare in healthy tissues.

## Targeting Protein Networks and Chromatin Re-Modelers

In addition to TFs, it is important to consider the role played by chromatin modulators in driving transformation. Mutated protein members “hijack” remodeling machinery to localize in different areas of the genome, leading to aberrant gene expression. Although TFs in cancer are undoubtedly important, open chromatin is more likely to facilitate gene expression. Therefore, the targeting of PPIs specific to oncogenically activated chromatin modulators may offer a more viable method to silence dysregulated transcription in cancer. One clear example is the BAF or mSWI/SNF complex, where genes encoding subunits or associated proteins are mutated in over 20% of cancers (Pierre and Kadoch, 2017). Such a high frequency of mutations correlated with specific oncogenic phenotypes can be attributed to a high degree of genetic non-redundancy within the complex. An example is SMARCB1 inactivation in early pediatric rhabdoid tumors, which is considered the sole genetic driving event in an otherwise genomically stable malignancy (Wang et al., 2017). Such stability is indicative of epigenetic changes caused by an oncogenically activated BAF complex. The loss of SMARCB1 reduces levels of the BAF complex, impairing normal function and transcriptional homeostasis. Subsequent alteration in genome-wide targeting reduces BAF binding to typical enhancers required for transcription of cell differentiation genes. Instead, remaining SMARCB1-deficient complexes maintain binding at super enhancers, causing preferential transcription of genes required for current cell identity maintenance, which may be due to higher affinity BAF complex binding at these sites. When specific proliferative progenitor cells are affected, cells are effectively locked into a highly proliferative and lowly differentiated state due to impaired enhancer targeting working to drive oncogenic transformation (Wang et al., 2017). Expression of another subunit of the complex, ARID1A, is lost in colon cancer in mice causing a similar reduction in levels of the BAF complex (Mathur et al., 2017). This causes its absence at thousands of enhancers and subsequent reduction/change in gene expression. ARID1B has a similar binding preference and can compensate to some extent by binding in the place of ARID1A, but the presence of this altered complex causes extensive dysregulation of gene expression. This further highlights the importance of complex composition and the non-redundant nature of PPIs within this complex. There are therefore a great many potential targets for therapy within the BAF complex. Indeed, comprehensive understanding of the relationships between biochemistry and function must be reached in order to unlock their greatest potential.

Another emerging field for PPI targeting is the modulation of the ubiquitin pathway. For example, proteasome-mediated degradation can be biased toward the preferential break down of tumor suppressors and the apparent preservation of oncoproteins in cancer cells (Wertz and Wang, 2019). As the process is a cascade, there are several proteins which offer valuable targets for anticancer therapies. There are three classes of enzymes responsible for ubiquitination, E1, E2, and E3, which comprise 2, 40, and over 600 isozymes in humans respectively (Li et al., 2008; Deshaies and Joazeiro, 2009; Schulman and Wade Harper, 2009). Although it has been possible to modulate the E1 and E2 members of the ubiquitination pathway, E3 enzymes have higher substrate specificity and offer greater potential for specific targeting. One of the most notable E3 PPIs for targeting is the MDM2:p53 interaction, where MDM2 is the negative regulator for p53 and therefore an important oncoprotein. Another method of modulation involving target proteins and E3 enzymes are Proteolysis targeting chimeras (PROTACs) (Sakamoto et al., 2001). PROTACs contain two moieties which independently bind a relevant target protein and an E3 ubiquitin ligase (Figure 2B). This brings the target into close proximity for ubiquitination by the E3 enzyme and marks the protein for degradation by the proteasome. This system has the advantage of being able to target proteins such as TFs, as PROTACs require only transient drug-target binding, whilst not inhibiting substrate activity. The ubiquitin pathway therefore offers an attractive therapeutic angle to the targeting of TFs, helping to modulate proteins which are otherwise difficult to mark.

It is clear that the wealth of existing proteomics data needs to be harnessed to address this area, looking at the specific interactions and phosphorylation states of putative TNBC oncogenes in disease contexts versus those observed in healthy tissue. This may take the form of targeted approaches using emerging techniques such as rapid immunoprecipitation mass spectrometry of endogenous proteins (RIME) (Mohammed et al., 2016) or co-immunoprecipitation coupled with mass spectrometry for the identification of PPIs and the analysis of post-translational modifications. However, unbiased and high-throughput approaches to investigate interactions and post-translational modifications in a whole-cell format are still lacking and therefore knowledge of particular TNBC oncoproteins is currently required to take this approach. The emerging field of single cell proteomics may offer the opportunity to perform unbiased screens to correlate particular protein states with cellular phenotypes in the future but, as of yet, high-throughput single-cell proteomics methods are not available for this purpose (Marx, 2019). However, the rate with which the single cell field is progressing bodes well for this technology and no doubt its development will offer unprecedented insight into PPIs driving malignancy. The combination of the above approaches may provide new therapeutic angles for the development of novel, more targeted and more effective TNBC therapies, as well as providing valuable insights into the mechanism of TNBC pathogenesis.

## Author Contributions

MW wrote the first draft of the manuscript and produced the figures. MW and RU wrote sections of the manuscript. All authors contributed to the manuscript revision, read, and approved the submitted version.

## Conflict of Interest

The authors declare that the research was conducted in the absence of any commercial or financial relationships that could be construed as a potential conflict of interest. The handling Editor declared a past co-authorship with one of the authors WK.
